# Involvement of APOBEC3A/B Deletion in Mouse Mammary Tumor Virus (MMTV)-like Positive Human Breast Cancer

**DOI:** 10.3390/diagnostics13061196

**Published:** 2023-03-22

**Authors:** Nathália de Sousa Pereira, Glauco Akelinghton Freire Vitiello, Marla Karine Amarante

**Affiliations:** 1 Oncology Laboratory, Department of Pathology, Clinical and Toxicological Analyses, Health Sciences Center, Londrina State University, Londrina 86057-970, PR, Brazil; 2 Translational Immuno-Oncology Group, International Research Center, A.C. Camargo Cancer Center, São Paulo 01508-010, SP, Brazil

**Keywords:** breast cancer, mouse mammary tumor virus, genetic polymorphism, clinicopathological parameters, APOBEC

## Abstract

The association between mouse mammary tumor virus (MMTV)-like sequences and human breast cancer (BC) is largely documented in the literature, but further research is needed to determine how they influence carcinogenesis. APOBEC3 cytidine deaminases are viral restriction factors that have been implicated in cancer mutagenesis, and a germline deletion that results in the fusion of the APOBEC3A coding region with the APOBEC3B 3′-UTR has been linked to increased mutagenic potential, enhanced risk of BC development, and poor prognosis. However, little is known about factors influencing APOBEC3 family activation in cancer. Thus, we hypothesized that MMTV infection and APOBEC3-mediated mutagenesis may be linked in the pathogenesis of BC. We investigated *APOBEC3A/B* genotyping, MMTV-like positivity, and clinicopathological parameters of 209 BC patients. We show evidence for active APOBEC3-mediated mutagenesis in human-derived MMTV sequences and comparatively investigate the impact of *APOBEC3A/B* germline deletion in MMTV-like *env* positive and negative BC in a Brazilian cohort. In MMTV-like negative samples, *APOBEC3A/B* deletion was negatively correlated with tumor stage while being positively correlated with estrogen receptor expression. Although *APOBEC3A/B* was not associated with MMTV-like positivity, samples carrying both MMTV-like positivity and APOBEC3A/B deletion had the lowest age-at-diagnosis of all study groups, with all patients being less than 50 years old. These results indicate that APOBEC3 mutagenesis is active against MMTV-like sequences, and that *APOBEC3A/B* deletion might act along with the MMTV-like presence to predispose people to early-onset BC.

## 1. Introduction

Breast cancer (BC) can be considered a major public health issue due to the number of women affected and the number of deaths it causes each year. In 2020, there were an estimated 2.3 million new cases of BC worldwide, which is equivalent to 24.5% of all cancers in women excluding non-melanoma skin cancers. Breast cancer is the fifth leading cause of death from cancer worldwide, accounting for 685,000 deaths in 2020 [1]. In Brazil, the estimate of new cases of BC predicts about 73,000 cases for the triennium 2023 to 2025, which corresponds to an estimated risk of 66.54 new cases per 100,000, positioning it as the most common type of cancer in the country. In terms of mortality, there were 17,825 deaths from BC in Brazil in 2020, which represents a risk of 16.47 deaths per 100,000 women [2].

Molecular subtypes in BC can be identified by the immunohistochemistry analysis of four key markers in the clinical routine: estrogen receptors (ER), progesterone receptors (PR), type 2 human epidermal growth factor receptor (HER2), and the cellular proliferation index Ki-67. These markers enable the identification of distinct molecular subtypes, each associated with prognostic factors and specific therapeutic targets [3], namely: luminal A (ER/PR^+^ HER2^−^), luminal B (ER/PR^+^ HER2^+^ or ER/PR^+^ HER2^−^ Ki67^medium/high^), HER2-enriched (ER^−^ PR^−^ HER2^−^), and triple-negative (ER^−^ PR^−^ HER2^−^) [3,4,5].

Despite having a good prognosis when detected in the early stages and treated in a timely fashion, BC is often detected in advanced stages in low- and middle-income countries, leading to a significant decrease in patient survival. The most important risk factor for BC is age over 50 years. Other risk factors are associated with hormonal or reproductive conditions, such as nulliparity, late pregnancy, less breastfeeding; with behavioral factors, such as obesity, alcohol consumption, physical inactivity; with work or occupation, such as night work and radiation; and genetic and hereditary conditions (5 to 10% of cases) [2,6,7,8,9,10].

At least 12% of all human tumors have a viral etiology, with human papilloma virus (HPV), Epstein–Barr virus (EBV), herpesvirus associated with Kaposi’s sarcoma (KSHV), hepatitis B virus (HBV), hepatitis C virus (HCV), the Merkel cell polyomavirus (MCPyV), and the human T-cell lymphotropic virus type 1 (HTLV-1) being prominent viruses associated with human cancers [11,12]. Viruses can contribute to oncogenesis in multiple ways, such as: (a) by directly encoding oncogenic proteins, (b) by causing chronic inflammation, and (c) by inducing genomic instability or other alterations in the host cells [12]. 

Initial research conducted by Bittner linked the mouse mammary tumor virus (MMTV), a virus from the *Betaretroviridae* genus of the *Retroviridae* family, to the development of BC in mice [13]. Since that revelation, scientists have been intrigued by the hypothesis that a retrovirus homologous to MMTV may play a role in the development of human BC. This interest was reignited when a complete proviral sequence 95% homologous to the MMTV genome was identified in human BC tissue samples, receiving the name of MMTV-like [14]. The literature has provided evidence over the years of the MMTV-like sequence’s role in the development of human BC [15,16]. However, the possible mechanisms behind MMTV-like association with BC remain unknown. 

Viruses are potent immune activators, and cancers associated with viral infections show signs of enhanced immunogenicity, presenting higher immune infiltrates, better prognosis, and enhanced response to immunotherapies with immune checkpoint inhibitors [17]. Mechanistically, viral-derived nucleic acids are recognized by intracellular innate immune sensors, such as Toll-like receptors (TLRs), RIG-like receptors (RLRs), and cyclic GMP–AMP synthase (cGAS)-STING, which trigger the production of type I and type III interferons. On target cells, IFNs-I and -III induce a so-called “antiviral state” characterized by cell cycle arrest, apoptosis, and increased immunogenicity. This effect is mediated by the induction of a large number of genes, known collectively as interferon-stimulated genes (ISGs) [18]. 

Among these, the apolipoprotein B mRNA-editing enzyme catalytic polypeptide-like 3 (APOBEC3) family, which in humans includes seven related cytidine deaminases (APOBEC3A, B, C, D, F, and G), play important roles in inactivating viruses by converting cytosine to uracil in TC dinucleotides of viral genomes, generating inactivating mutations hampering their replication [19]. On the other hand, it has been shown that some of these enzymes are also capable of generating point mutations in the host cell genome as a side effect, potentially leading to malignant transformation [20].

Indeed, mutations caused by enzymes from the APOBEC3 family have been detected in a great proportion of tumors from diverse cancer types [19], such as bladder, cervix, lung, head and neck, and BC [21,22], and APOBEC3-mediated mutations are thought to be responsible for the COSMIC single base signatures (SBS) 2 and 13 [19]. These mutations likely influence tumor evolution through the activation of oncogenes and inactivation of tumor suppressor genes [20], and in-depth characterization of this mechanism has highlighted APOBEC3A and APOBEC3B as the main mutagens in this context [23,24]. More recently, it has been shown that APOBEC3A is a more potent mutagenic agent than APOBEC3B, and that APOBEC3A signatures are far more frequent in human cancers than APOBEC3B-attributed mutations [25].

A germline copy number deletion polymorphism has been identified that results in the APOBEC3B coding sequence being deleted and its 3′ untranslated region (3′-UTR) being linked to the coding exons of APOBEC3A, thus, creating a hybrid gene (*APOBEC3A/B*, Gene ID on NCBI databank: 100913187). *APOBEC3A/B* has already been associated with a greater burden of mutational signatures consistent with APOBEC3 activity and with an increase in the risk of several types of cancer, including BC [26,27,28,29,30], suggesting that carriers of the deleted allele had greater APOBEC3A activity, since the deletion generates a more stable mRNA isoform for APOBEC3A [24]. However, it is interesting to point out that the *APOBEC3B* deletion polymorphism has also been associated with reduced risk for the development of certain types of cancer, such as lung cancer and ovarian cancer, in recent works in the literature [31,32]. Moreover, both APOBEC3A and APOBEC3B display cytidine-deaminase-independent roles in carcinogenesis that add complexity when it comes to comprehending their roles in cancer [33,34,35]. Thus, the association of *APOBEC3A/B* genotypes with cancer development remains controversial.

Despite the well-documented importance of APOBEC3 family enzymes in the carcinogenesis of several tumors, the mechanisms that regulate their activity in tumor development of BC remain poorly explored in the literature. Likewise, the possible oncogenic mechanism of MMTV-like in BC remains unknown, despite its current association with human BC. Furthermore, previous works have shown that HPV-associated cancers are associated with a APOBEC3 mutational signature and *APOBEC3A/B* deletion [20,36], and that APOBEC3-mediated mutations are present in HPV genomes [37,38]. Based on recent suggestions proposed by groups that have been assessing the association of MMTV-like with BC for years [15,39,40], who hypothesized that MMTV-like and APOBEC3-mediated mutagenesis may work together to cause BC oncogenesis, we sought to investigate how *APOBEC3A/B* and MMTV-like *env* sequences influence BC susceptibility and clinical presentation.

## 2. Materials and Methods

### 2.1. Study Subjects and Data

For the present study, we used data from overlapping BC cohorts derived from two previous studies by our group investigating MMTV-like prevalence and *APOBEC3A/B* association with BC susceptibility [40,41]. The patients were being treated at Londrina Cancer Hospital (LCH) under the Brazilian Public Health System (SUS). All participants signed a form of freely informed consent before the study could proceed and the experimental design was approved by the Human Ethics Committee of Londrina State University (CAAE 73557317.0.0000.5231 and CAAE 47709015.2.0000.5231). 

From the patients’ medical records, the following clinicopathological features were obtained: age at diagnosis, tumor size, histopathological grade, lymph node metastasis (LNM) status, pathological disease stage, and immunostaining for Ki67, tumor suppressor p53, ER, PR, and HER2. The Laboratory of Clinical Pathology of the LCH conducted immunostaining analysis as a routine clinical procedure following established protocols [42,43]. The Union of International Control of Cancer (UICC) classification criteria were adopted to determine clinical stage of BC [44]. BC was classified according to the number of tumor cells expressing Ki67 in low (15%), intermediate (16–30%), or high (>30%) expression groups [45]. 

In total, 209 patients with data for *APOBEC3A/B* genotyping, MMTV-like detection in BC tissue, and clinicopathological parameters were included in the present analyses. Median age of the patients was 55 (interquartile range: 47–66) years. Median tumor size was 2.2 (interquartile range: 1.5–3.2) cm, with 46.9% of the sampled presenting a tumor ranging from 1.51–3.00 cm. Regarding the histological type, the majority of tumors were classified as invasive ductal carcinoma (90.3%). Considering the specific subtypes of BC, data from the medical records of 195 patients were available and most were classified as luminal A (68.7%), followed by luminal B-HER2^+^ (12.3%), triple negative (TN) (12.3%), and HER2 overexpressed (6.7%). 

### 2.2. Detection of MMTV-Like env Gene and APOBEC3A/B Genotyping

*APOBEC3A/B* genotyping was performed by allele-specific PCR [41], while the detection of MMTV-like *env* in BC tissue samples was performed using a nested PCR protocol [40]. For 35 patients, raw Sanger-sequencing data for the inner nested PCR-amplified MMTV-like *env* sequence were also available and chromatograms were inspected using the BioEdit7 software. Human-derived MMTV sequences were also retrieved from NCBI nucleotide databank and aligned using the MUSCLE algorithm in the MEGA 7 software [46]. Phylogeny was analyzed through the maximum likelihood method [47].

Of the 209 BC patients included in this study, 40 were positive for MMTV-like *env* gene and 169 were negative. Of those 40 MMTV-like *env* positive cases, 26 were classified as luminal A, 6 as luminal B, 5 as HER2 overexpressed, and 1 as TN. The *APOBEC3A/B* genotypes observed in the analyzed samples were homozygosis for the wild genotype (WT/WT) and the heterozygous genotype (WT/Del), while no sample with the homozygous genotype (Del/Del) was found. WT/WT genotype was observed in 86.1% (180/209) of BC samples and WT/Del genotype was observed in 13.9% (29/209) patients.

### 2.3. Statistical Analyses

Kendall’s rank correlation test was applied for correlation analyses and a chi-square test was employed to compare *APOBEC3A/B* genotypes frequency between MMTV-like positive and negative patients. To compare age-at-diagnosis among patients stratified by the presence of MMTV-like *env* gene and *APOBEC3A/B* deletion (4 groups), Kruskal–Wallis test was applied followed by Dunn’s post-test. The SPSS 20.0 (SPSS Inc., Chicago, IL, USA) and GraphPad Prism version 8.0 (GraphPad Software Inc., San Diego, CA, USA) were used for all data analyses, applying two-tailed tests with significance level set at 0.05.

## 3. Results

To investigate whether APOBEC3-mediated mutagenesis operates in MMTV-like *env* positive BC, we downloaded human-derived MMTV-like *env* sequences from the NCBI nucleotide databank that corresponded to the inner fragment of the nested PCR amplicon used in most studies to detect MMTV-like sequences in human samples. For this analysis, the corresponding sequence stretch from the MMTV reference genome was aligned to the nucleotide bank using the BLAST algorithm, and sequences corresponding to human isolates with >95% of query cover were downloaded and aligned through the MUSCLE algorithm, resulting in an alignment window of 245 base pairs. Then, aligned sequences were inspected for TC > TT and GA > AA variations as putative APOBEC3-driven mutations. 

**Figure 1:**
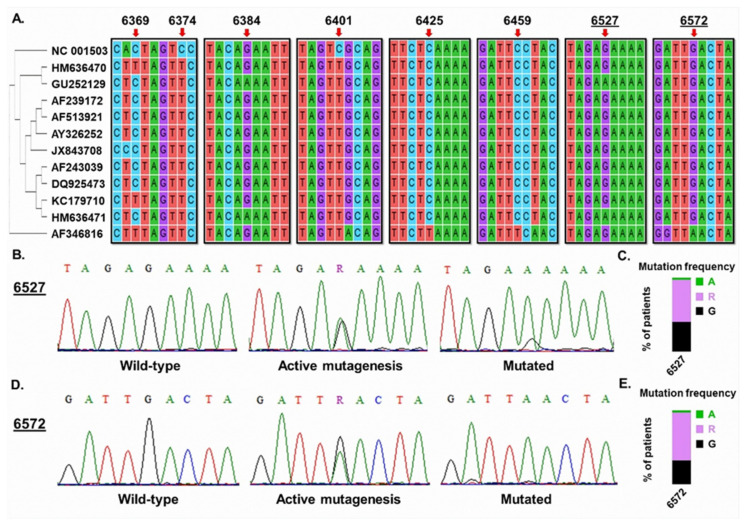
**Evidence for APOBEC3-mediated editing in human-derived MMTV sequences.** (**A**). APOBEC3 mutation sites in human-derived MMTV-env sequences from NCBI Nucleotide databank. Numbers indicate nucleotide position in the reference MMTV genome (NC_001503). (**B**). Sanger-sequencing chromatograms for nucleotide 6527 of MMTV-like genome representing a wild-type sample (**left**), a sample with ongoing mutagenesis in viral genome (**middle**), and a sample with consolidated mutation (**right**). (**C**). Frequency of patients showing genotypes represented in (**B**). (**D**). Sanger-sequencing chromatograms for nucleotide 6572 of MMTV-like genome representing a wild-type sample (**left**), a sample with ongoing mutagenesis in viral genome (**middle**), and a sample with consolidated mutation (**right**). (**E**). Frequency of patients showing genotypes represented in (**D**).

In total, eight such mutation sites were annotated (Figure 1A), including two in TCA or TGA sites (positions 6425 and 6572 of MMTV reference genome, respectively), which are indicative of APOBEC3A or APOBEC3B activity (Figure 1A). Then, Sanger-sequencing chromatograms for 35 samples in our cohort were inspected at these sites to investigate if there was evidence of active APOBEC3-mediated mutagenesis in MMTV-like *env* sequences derived from human samples. Although we found evidence of consolidated mutations for most of these sites, for nucleotides 6527 and 6572 we found 57% and 64% of samples, respectively, showing signal for both A and G (R) in Sanger chromatograms (Figure 1B–E).

Interestingly, these mutations occurred in sites that did not alter the amino acid in protein (6527: GAG > GAA, both coding for glutamine; 6572: TTG > TTA, coding for leucine), which may explain their high frequency in the analyzed samples. Importantly, these samples were sequenced in duplicate in both forward and reverse orientation, allowing for reliable confirmation of such alterations. Therefore, these data indicate that APOBEC3-driven mutations are sources of genetic variation in human-derived MMTV sequences and that this process is active in BC tissue, generating intra-host variability in viral sequences.

Next, we sought to evaluate the possible impact of *APOBEC3A/B* deletion in MMTV-like *env* positive BC. We started by investigating the association between *APOBEC3A/B* deletion genotypes and the presence of MMTV-like *env* in BC. No significant difference is observed between the *APOBEC3A/B* deletion frequency in MMTV-like *env* positive and MMTV-like *env* negative BC samples, indicating that it is not associated with MMTV-like positivity in our BC cohort (Table 1).

**Figure 2:**
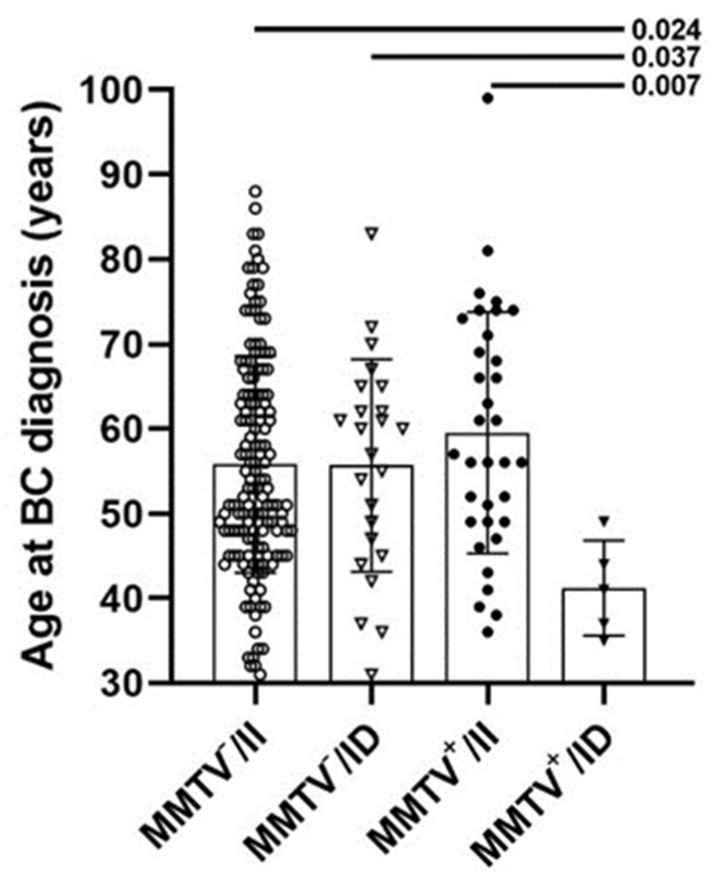
**Age comparison between patients’ groups defined by *APOBEC3A/B* deletion and MMTV-like positivity.** Patients carrying both MMTV-like *env* positivity and *APOBEC3A/B* deletion (MMTV+/ID) have the lowest age compared to other groups. Each point represents a single patient. Bars represent mean and standard deviation. *p*-values for Dunn’s post-test are shown.

Then, the impact of *APOBEC3A/B* deletion on BC clinical presentation in MMTV-like negative and MMTV-like positive BC was evaluated (Table 2). Considering the MMTV-like *env* negative BC samples, *APOBEC3A/B* deletion is negatively correlated with tumor stage (Tau-c = −0.151; *p* = 0.044), while in MMTV-like *env* positive BCs, *APOBEC3A/B* deletion positively correlates with ER (Tau-b = 0.179; *p* = 0.041) and shows a strong negative correlation with patients age-at-diagnosis (Tau-c = −0.382; *p* = 0.009). Indeed, patients presenting both MMTV-like *env* and *APOBEC3A/B* deletion have the lowest age-at-diagnosis of the cohort (41 years old on average) when compared to all other groups, with all samples being less than 50 years old (Figure 2). Interestingly, despite the young age at diagnosis, these patients had luminal BCs (four patients with luminal A and one patient with luminal B), which correspond to usually later-onset BC subtypes when compared to TN and HER2-overexpressed BCs.

## 4. Discussion

The variability of MMTV-like ***env*** gene presence in BC tumor tissue across the world ranges from 0 to 74% of samples examined [15]. In Brazil, in a previous study conducted by our research group, we observed the presence of MMTV-like ***env*** sequence in 18.9% of tumor tissue samples. Surprisingly, we found some correlations between the presence of MMTV-like sequences with clinicopathological parameters in the luminal-B and HER2-overexpressed BC subtypes considered to predict a better disease prognosis, such as smaller tumor size, lower TNM staging, and lower frequency of lymph node metastases [40]. This led us to hypothesize that MMTV-like could initiate antiviral immune responses within the tumor microenvironment, as increased expression of genes related to viral infection response, such as interferons (IFNs), had already been observed in BC [48]. 

The family of APOBEC3 enzymes are activated by type I IFNs and act on viral restriction, by inducing mutations in the virus genome [49]. However, the mutagenic process promoted by these enzymes may also affect the host’s genomic DNA. Recently, multiple tumor types have revealed that dysregulation of APOBEC3 is a significant factor in tumor mutations, resulting in intratumor heterogeneity, clonal evolution, and tumor adaptation to therapy [50]. Also, studies demonstrated a relationship between viral infection, increased expression of APOBEC3, and the mutagenic signature of this enzyme in several types of cancer associated with viruses, such as HPV-positive cervical and oropharynx cancers [51] and EBV-positive gastric cancer [52]. 

Several studies already demonstrated the role of APOBEC3 in the infection by MMTV in mice and in the development of BC in these animals. In a study conducted by Okeoma et al. [53], it was found that APOBEC3 reduces infection by MMTV in its natural host, by limiting infection in dendritic cells. Thus, increased levels of APOBEC3 in mice can lead to enhanced viral restriction [53,54,55]. Further, a recent study has shown that the MMTV provirus carries a lesser number of mutations caused by APOBEC3 enzymes compared to lentiviruses, such as human immunodeficiency virus (HIV). Apparently, MMTV reverse transcriptase has evolved, increasing its enzymatic speed, in order to avoid the access of APOBEC3s to DNA strands exposed during reverse transcription [56]. Here, we show evidence for active APOBEC3-mediated mutations in MMTV-like sequences derived from human samples. This result is concordant with previous work showing that viral sequences of MMTV replicating in human cells in vitro are enriched for APOBEC3-mediated mutational signatures [57].

The *APOBEC3A/B* germline deletion has been highlighted as a susceptibility marker for the development of BC in Asian and Caucasian populations [58], but a previous study from our research group suggests that there is no association between *APOBEC3A/B* genotypes and BC susceptibility in the Brazilian population [41]. Other studies show that the loss of the *APOBEC3B* gene can increase susceptibility to viral infection, as in infections by HIV, HBV, and HPV [59,60,61,62]. Interestingly, in the study from Zhang [62], in addition to the increased susceptibility to HBV infection, a greater susceptibility to the development of hepatocellular carcinoma associated with HBV was also observed. In our study, no association was found between *APOBEC3A/B* genotypes and MMTV-like positivity in BC patients. However, interesting findings were noted regarding the association between *APOBEC3A/B* polymorphism and clinicopathological features in MMTV-positive patients.

Correlation analysis between *APOBEC3A/B* genotypes and clinicopathological parameters of tumor samples positive for the presence of MMTV-like *env* gene demonstrate a correlation between the heterozygous genotype and lower age-at-diagnosis. In a study conducted by Gansmo et al. [31], *APOBEC3A/B* was also associated with lower age-at-diagnosis in patients with lung and prostate cancer; however, this association was not found when evaluating patients with BC and colon cancer. In agreement with our findings, Cescon et al. [63] found that age-at-diagnosis is slightly lower in BC patients that carry the deleted allele. Strikingly, in our study, patients presenting simultaneous positivity for MMTV-like *env* and *APOBEC3A/B* deletion have significantly lower age-at-diagnosis, with all patients being less than 50 years old, despite bearing luminal tumors, which are classically diagnosed at ages above 50. 

These results may suggest that patients with *APOBEC3A/B* deletion in the presence of MMTV-like sequences in the tumor microenvironment may have even greater genetic instability and mutational rates when compared to patients presenting only one of these factors, which may result in earlier development of BC. It is known that insertion of viral sequences may cause insertional mutagenesis in the host cell while also inducing the expression of APOBEC3 clusters through IFN signaling; further, while APOBEC3A and APOBEC3B pose mutagenic potential for host cells per se, patients presenting the *APOBEC3A/B* deletion are described as having a greater APOBEC3-mediated mutational burden [26,29], reasonably explaining the mechanisms by which these factors may synergize to predispose patients to early-onset BC.

Also, we observed a correlation with the expression of ER in MMTV-like positive tumor tissue. It has been suggested that the APOBEC3B expression is induced by estradiol in ER+ BC cell lines, suggesting that APOBEC3B is an estrogen-responsive gene while APOBEC3A is not [64]. In addition, Periyasamy et al. [65] show that APOBEC3B is required for the regulation of gene expression by ER and acts by causing deamination at ER binding regions. They demonstrate that APOBEC3B expression is associated with poor patient survival in ER+ BC, reinforcing the physiological significance of these enzymes for ER action. Besides this association with worse prognosis, recent studies demonstrate that patients with hormone-receptor-positive and HER2-negative BC have a higher resistance to hormonal treatment due to the action of enzymes from the APOBEC3 family. This is due to the accumulation of APOBEC3-induced mutations in *PIK3CA* and other alterations at non-hotspot locations, which results in metastatic and endocrine-resistant BC [66,67]. An interesting finding pointed out by André et al. [68] is that these patients, due to having the PI3K pathway activated, can benefit from inhibitors of this pathway that have been recently introduced into clinical practice [66].

In addition, we could observe that patients that were negative for MMTV-like DNA and with a heterozygous genotype for *APOBEC3A/B* had a lower tumor stage compared to patients with a wild genotype. Otherwise, Rezaei et al. [69] investigated the association between *APOBEC3A/B* genotypes and clinicopathological parameters in Iranian BC patients, including tumor stage, and no significant association was observed, suggesting that the influence of *APOBEC3A/B* in BC may vary among different populations with differing genetic background and exposed to different risk factors.

## 5. Conclusions

In conclusion, the present study shows that APOBEC3-mediated mutagenesis occurs in MMTV-like ***env*** sequences from BC patients, but *APOBEC3A/B* genotypes are not associated with the presence of MMTV-like DNA. Interestingly, as shown previously in the literature, *APOBEC3A/B* genotypes are correlated with clinicopathological parameters of BC patients, and we further show that it may also synergize with MMTV-like sequences to predispose patients to early-onset BC. As the contributions of MMTV-like sequences, as well as of *APOBEC3A/B*, to the development of BC have been explored worldwide, varying according to the population analyzed, further studies should be carried out to evaluate the interaction of APOBEC3 enzymes and MMTV-like sequences in patients with breast carcinoma around the world.

## Figures and Tables

**Table 1:** Association between *APOBEC3A/B* genotypes and detection of MMTV-like *env* gene in BC samples.

*APOBEC 3A/B* Genotypes	MMTV-like *env*			
	Negative	Positive	χ^2^ *p*-Value	Odds Ratio (CI 95%)
WT/WT [*n* (%)]	145 (85.8)	35 (87.5)	1.00	0.87 (0.31–2.46)
WT/Del [*n* (%)]	24 (14.2)	5 (12.5)		

χ^2^: chi-squared test; CI: confidence interval.

**Table 2:** Data on the clinicopathological characteristics of the patients.

Parameter	MMTV-like Positive (*n* = 40)			MMTV-like Negative (*n* = 169)		
*APOBEC3A/B*	WT/WT	WT/Del	Tau (*p*)	WT/WT	WT/Del	Tau (*p*)
**Age (years)**						
Mean (SD)	60 (14)	41 (6)	−0.382 (0.009 *)	56 (13)	56 (13)	0.007 (0.906)
Median (IQR)	57 (22)	41 (7)		53 (17)	59 (18)	
<40 [*n* (%)]	3 (8.8)	2 (40.0)		13 (8.9)	3 (12.5)	
40–49 [*n* (%)]	7 (20.6)	3 (60.0)		41 (28.3)	5 (20.8)	
50–59 [*n* (%)]	8 (23.5)	0 (0.0)		36 (24.8)	4 (16.6)	
60–69 [*n* (%)]	7 (20.6)	0 (0.0)		33 (22.7)	9 (37.5)	
70–79 [*n* (%)]	7 (20.6)	0 (0.0)		16 (11.1)	2 (8.3)	
≥80 [*n* (%)]	2 (5.9)	0 (0.0)		6 (4.2)	1 (4.3)	
Missed [*n* (%)]	1			0		
**Tumor size (cm)**						
Mean (SD)	2.9 (2.4)	3.7 (1.7)	0.192 (0.186)	2.8 (1.9)	2.6 (1.7)	−0.006 (0.918)
Median (IQR)	2.0 (0.7)	3.5 (1.5)		2.2 (2.0)	2.4 (1.4)	
0–1.5 [*n* (%)]	6 (17.1)	1 (20.0)		41 (28.7)	6 (25.0)	
1.51–3.0 [*n* (%)]	21 (60.0)	1 (20.0)		62 (43.3)	14 (58.4)	
>3.0 [*n* (%)]	8 (22.9)	3 (60.0)		40 (28.0)	4 (16.6)	
Missed	1			2		
**Histopathological grade [*n* (%)]**						
I	5 (15.1)	0 (0.0)	0.091 (0.504)	22 (16.1)	1 (4.8)	0.043 (0.534)
II	11 (33.4)	2 (40.0)		57 (41.6)	11 (52.4)	
III	17 (51.5)	3 (60.0)		58 (42.3)	9 (42.8)	
Missed	2			11		
**Tumor stage [*n* (%)]**						
0	2 (7.7)	0 (0.0)	0.097 (0.394)	6 (5.3)	2 (10.0)	−0.151 (0.044 *)
I	8 (30.8)	0 (0.0)		22 (19.3)	5 (25.0)	
II	7 (26.9)	3 (75.0)		46 (40.4)	11 (55.0)	
III	7 (26.9)	1 (25.0)		34 (29.7)	1 (5.0)	
IV	2 (7.7)	0 (0.0)		6 (5.3)	1 (5.0)	
Missed	10			38		
**Lymph node metastasis [*n* (%)]**						
Positive	16 (47.1)	2 (40.0)	−0.047 (0.765)	68 (49.3)	7 (30.4)	−0.132 (0.085)
Negative	18 (52.9)	3 (60.0)		70 (50.7)	16 (69.6)	
Missed	1			8		
**Estrogen receptor [*n* (%)]**						
Positive	27 (79.4)	5 (100.0)	0.179 (0.041 *)	108 (78.3)	20 (86.9)	0.075 (0.276)
Negative	7 (20.6)	0 (0.0)		30 (21.7)	3 (13.1)	
Missed	1			8		
**Progesterone receptor [*n* (%)]**						
Positive	19 (55.9)	2 (40.0)	−0.107 (0.511)	74 (53.6)	13 (56.5)	0.020 (0.796)
Negative	15 (44.1)	3 (60.0)		64 (46.4)	10 (43.5)	
Missed	1			8		
**HER2 [*n* (%)]**						
Positive	10 (30.3)	1 (20.0)	−0.077 (0.605)	24 (17.9)	2 (8.7)	−0.088 (0.182)
Negative	23 (69.7)	4 (80.0)		110 (82.1)	21 (91.3)	
Missed	2			12		
**Ki67 [*n* (%)]**						
Low	9 (29.0)	0 (0.0)	0.198 (0.160)	36 (28.8)	2 (10.5)	0.023 (0.713)
Intermediate	13 (42.0)	2 (50.0)		48 (38.4)	13 (68.4)	
High	9 (29.0)	2 (50.0)		41 (32.8)	4 (21.1)	
Missed	5			25		
**p53 [*n* (%)]**						
Positive	11 (36.7)	3 (75.0)	0.251 (0.173)	45 (33.8)	5 (23.8)	−0.073 (0.331)
Negative	19 (63.3)	1 (25.0)		88 (66.2)	16 (76.2)	
Missed	6			15		

*: *p* < 0.05; SD: standard deviation; IQR: interquartile range; IDC: invasive ductal carcinoma; ILC: invasive lobular carcinoma.

## Data Availability

The data are not publicly available due to ethical reasons.
